# Assessment of the Prevalence and Trajectory of Depressive Symptoms by Sexual Orientation During Physician Training

**DOI:** 10.1001/jamahealthforum.2022.0812

**Published:** 2022-04-29

**Authors:** Tejal H. Patel, Jennifer L. Cleary, Zhuo Zhao, Katherine E. T. Ross, Srijan Sen, Elena Frank

**Affiliations:** 1 Michigan Neuroscience Institute, University of Michigan, Ann Arbor; 2 Department of Psychology, University of Michigan, Ann Arbor; 3 Eisenberg Family Depression Center, University of Michigan, Ann Arbor

## Abstract

This cohort study uses survey data to assess the prevalence and development of depressive symptoms among sexual minority and heterosexual physicians during residency training.

## Introduction

In the general population, sexual minority individuals experience higher rates of depression compared with their heterosexual peers.^1^ Resident physicians also have a high risk of depression. Although sexual minority trainees experience higher rates of mistreatment and other unique stressors, little is known about their mental health.^2,3,4,5^ Here, we assess the prevalence and development of depressive symptoms among sexual minority and heterosexual physicians over the course of residency training.

## Methods

The University of Michigan Institutional Review Board approved this cohort study. All participants provided informed consent during online survey completion and were compensated between $50 and $125. The study followed the AAPOR reporting guideline for survey studies.

Incoming interns from 14 specialties across 537 residency institutions who enrolled in the Intern Health Study completed surveys 2 months before starting residency and quarterly throughout the internship year in 2016, 2017, or 2018. The surveys assessed depressive symptoms using the Patient Health Questionnaire-9 (PHQ-9).^6^ On the preinternship survey, sexual orientation response options included heterosexual, gay or lesbian, bisexual, other, and prefer not to say. Given the small proportion of trainees who self-identified as a sexual minority, we combined gay or lesbian, bisexual, and other into a single sexual minority category (eTable 1 in the Supplement).

To identify differences in trajectories of depressive symptoms throughout the internship year, we fit a linear mixed model with random effects for within-person dependence and fixed effects for sexual orientation and known covariates (sex, age, specialty, work hours, sleep hours, and survey time point, which was analyzed categorically). Higher scores indicated greater depressive symptoms. We used R version 3.5.1 to conduct the analysis. *P* values less than .05 were considered significant, and all tests were 2 tailed.

## Results

This study enrolled 8261 of 14 718 interns (56.1%) invited in 2016, 2017, and 2018. Of the 8261 participants, 7013 (84.9%) disclosed their sexual orientation, completed at least 1 quarterly follow-up survey, and were included in the analysis. Their mean (SD) age was 27.5 (2.6) years; there were 3652 women (52.1%) and 496 sexual minority individuals (7.1%) (Table).

**Table.** Characteristics of Study Participants

Characteristic	No. of participants (%)	
	Heterosexual (n = 6517)	Sexual minority (n = 496)
Age, y, mean (SD)	27.4 (2.5)	28.0 (2.9)
Sex		
Women	3420 (52.5)	232 (46.8)
Men	3093 (47.5)	262 (52.8)
Not reported	4 (0.1)	2 (0.4)
Specialty		
Nonsurgical	5391 (82.7)	406 (81.9)
Surgical	1113 (17.1)	90 (18.1)
Not reported	13 (0.2)	0
Self-reported weekly work hours, mean (SD)	63.5 (18.8)	63.7 (17.9)
Self-reported daily sleep hours, mean (SD)	6.9 (1.5)	6.9 (1.5)
PHQ-9 score > 10		
Baseline	244 (3.7)	27 (5.4)
Quarter		
1 (September)	1032 (15.8)	99 (20.0)
2 (December)	1033 (15.9)	107 (21.6)
3 (March)	939 (14.4)	105 (21.2)
4 (June)	852 (13.1)	99 (20.0)

Abbreviation: PHQ-9, Patient Health Questionnaire-9.

Sexual minority individuals entered the internship year with higher PHQ-9 scores than heterosexual participants, with a mean (SD) of 3.3 (3.3) vs 2.6 (3.0) at baseline and 7.1 (4.9) vs 5.7 (4.6) at quarter 4. Sexual minority interns also reported higher PHQ-9 scores compared with their heterosexual peers at all assessment points (Figure). Depressive symptoms increased at a greater rate throughout the year for sexual minority physicians compared with their heterosexual peers primarily in the second half of training, with β values of 0.48 (95% CI, 0.07-0.89; *P* = .02) and 0.52 (95% CI, 0.19-0.93; *P* = .01) in quarters 3 and 4, respectively.

**Figure.**
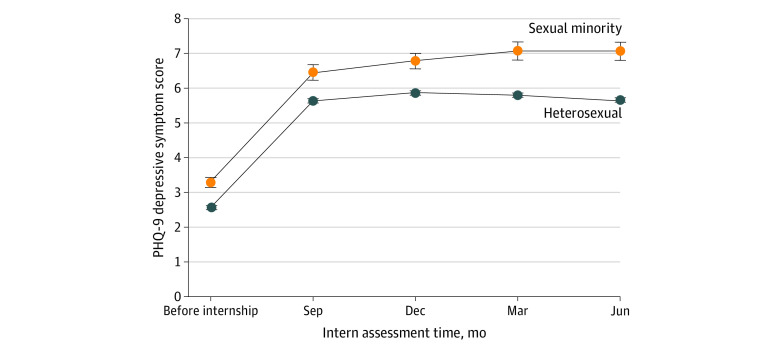
Depressive Symptoms Among Sexual Minority and Heterosexual Interns Assessed at Quarterly Intervals Using the Patient Health Questionnaire-9 Throughout the Internship Year Differences in mean Patient Health Questionnaire-9 (PHQ-9) depressive symptom scores among sexual minority and heterosexual interns were assessed at 3-month intervals before and throughout (September, December, March, and June) the internship year in 2016, 2017, and 2018. The trajectory of depressive symptoms during the second half of training (quarters 3 and 4) differed significantly between sexual minority and heterosexual physicians (quarter 3: β = 0.48 [95% CI, 0.07-0.89], *P* = .02; vs quarter 4: β = 0.52 [95% CI, 0.19-0.93], *P* = .01), even after accounting for specialty, sexual orientation, age, work hours, sleep hours, and sex.

## Discussion

Using data from a national longitudinal cohort study of US medical interns, our results suggest that sexual minority interns experience greater depressive symptoms than their heterosexual peers and that this gap widens over the course of training. In this study, depressive symptoms peaked at 6 months and then declined steadily for heterosexual interns, whereas depressive symptoms among sexual minority interns continued to increase throughout the year. These diverging trajectories may reflect additional stressors sexual minority physicians face, including high rates of harassment and discrimination and a lower sense of belonging, and may contribute to disparities in burnout, suicidal thoughts, and attrition.^2,3,4,5^

Our study has some limitations. Because of the sample size, it was not possible to assess different sexual minority subgroups separately or account for the effects of intersecting identities. In addition, gender identity information was not available and could not be factored into our model.

As our results suggest, further research is needed on the drivers of mental health disparities among sexual minority and heterosexual physician trainees. Targeted efforts are also needed to facilitate a healthier and more inclusive educational environment capable of developing and supporting a diverse physician workforce.

## Notes

Supplement. **eTable 1.** Survey Response Rates
