# Supplementary material for: Assessment of the Prevalence and Trajectory of Depressive Symptoms by Sexual Orientation During Physician Training
Source: JAMA Health Forum. 2022 Apr 29;3(4):e220812. doi: 10.1001/jamahealthforum.2022.0812 (PMC9055449; doi:10.1001/jamahealthforum.2022.0812)
Supplement: Supplement. — eTable 1. Survey Response Rates [file jamahealthforum-e220812-s001.pdf]

Supplementary Online Content

Patel TH, Cleary JL, Zhao Z, Ross KET, Sen S, Frank E. Assessment of the prevalence and trajectory of depressive symptoms by sexual orientation during physician training. *JAMA Health Forum*. 2022;3(4):e220812. doi:10.1001/jamahealthforum.2022.0812

eTable 1. Survey Response Rates

This supplementary material has been provided by the authors to give readers additional information about their work.

© 2022 Patel TH et al. *JAMA Health Forum*.

eTable 1. Survey Response Rates

Time Points	Heterosexual (N=7612)	Sexual minority (N=589)	Total (N=8201)
Baseline	7612 (100%)	589 (100%)	8201 (100%)
Quarter 1 (September)	5889 (77.4%)	454 (77.1%)	6343 (77.3%)
Quarter 2 (December)	5202 (68.3%)	411 (69.8%)	5613 68.4%)
Quarter 3 (March)	4892 (64.3%)	367 (62.3%)	5259 (64.1%)
Quarter 4 (June)	4489 (59.0%)	340 (57.7%)	4829 (58.9%)

© 2022 Patel TH et al. *JAMA Health Forum*.
